# Blockade of stress-induced increase of glutamate release in the rat prefrontal/frontal cortex by agomelatine involves synergy between melatonergic and 5-HT_2C _receptor-dependent pathways

**DOI:** 10.1186/1471-2202-11-68

**Published:** 2010-06-03

**Authors:** Daniela Tardito, Marco Milanese, Tiziana Bonifacino, Laura Musazzi, Massimo Grilli, Alessandra Mallei, Elisabeth Mocaer, Cecilia Gabriel-Gracia, Giorgio Racagni, Maurizio Popoli, Giambattista Bonanno

**Affiliations:** 1 Center of Neuropharmacology - Department of Pharmacological Sciences and Center of Excellence on Neurodegenerative Diseases, University of Milano, Italy; 2 Department of Exp. Medicine, Section of Pharmacology and Toxicology and Center of Excellence for Biomedical Research and National Institute of Neuroscience, University of Genova, Italy; 3 IDR Servier, Croissy-sur-Seine, France; 4 I.R.C.C.S San Giovanni di Dio - Fatebenefratelli, Brescia, Italy

## Abstract

**Background:**

Agomelatine is a melatonergic receptor agonist and a 5HT_2C _receptor antagonist that has shown antidepressant efficacy. In order to analyze separately the effect of the two receptorial components, rats were chronically treated with agomelatine, melatonin (endogenous melatonergic agonist), or S32006 (5-HT_2C _antagonist), and then subjected to acute footshock-stress.

**Results:**

Only chronic agomelatine, but not melatonin or S32006, completely prevented the stress-induced increase of glutamate release in the rat prefrontal/frontal cortex.

**Conclusions:**

These results suggest a potential synergy between melatonergic and serotonergic pathways in the action of agomelatine.

## Background

Despite the improved tolerability of newer antidepressants such as the selective serotonin reuptake inhibitors (SSRIs) [1-4] and the dual serotonin-norepinephrine reuptake inhibitors (SNRIs), adverse events associated with their use can influence adherence rates. There is an unmet need for a better understanding of the mechanisms and therapeutic management of depression, as well as for treatments with improved efficacy and tolerability. Indeed, both the pathophysiology of depression and the exact mechanisms whereby antidepressants elicit a therapeutic response have not been fully explored. An increasing body of evidence indicates that depression is associated with a disruption of circadian rhythms, suggesting that resetting of disrupted circadian rhythms may play a pivotal role in the treatment of this condition [5-11]. Agomelatine is a melatonergic receptor (MT1/MT2) agonist [12] and 5HT_2C _receptor antagonist [13] that has showed antidepressant efficacy in animal models [14-17] and clinical trials [18-21].

Recent findings have shown that, in addition to the changes induced in monoamine transmission, antidepressants may work by modulating glutamate release and transmission in relevant limbic and cortical areas [22-24]. In this regard, we have previously found that different chronic antidepressant treatments (including agomelatine) abolish the increase of depolarization-evoked glutamate release, induced by acute footshock (FS)-stress, from synaptosomes of prefrontal/frontal cortex (P/FC) [25,26]. Here we studied the two separate components in the mechanism of agomelatine, by treating rats chronically with either agomelatine, or melatonin (the endogenous agonist of MT1/MT2 receptors), or S32006 (a selective 5-HT_2C _antagonist), and then subjected the rats to FS-stress as above. Aim of the experiments was to investigate whether one of the two receptorial components was sufficient for the dampening action of agomelatine on stress-induced glutamate release, or both components were necessary. We found that chronic treatment with either melatonin or S32006 did not significantly reduce the stress-induced glutamate release, while chronic agomelatine treatment, as observed previously, completely abolished the stress-induced glutamate release, suggesting a potential synergy between MT1/MT2- and 5-HT_2C_-dependent pathways in the action of agomelatine.

## Results

### Depolarization-evoked release of glutamate from P/FC synaptosomes is increased after acute footshock stress

**Figure 1:**
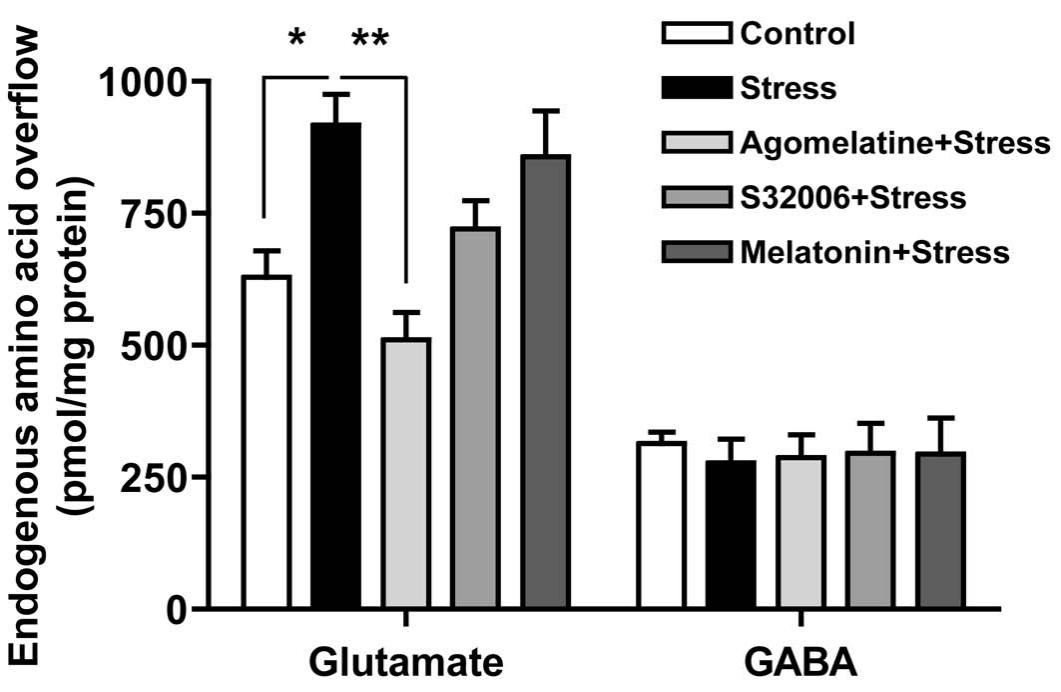
**Chronic agomelatine treatment but not melatonin or S32006 reduced stress-induced increase of glutamate release in the rat P/FC**. 15 mM KCl-evoked glutamate and GABA release in rats treated with vehicle (control), subjected to acute FS-stress (Stress), chronically treated with Agomelatine and then subjected to stress (Agomelatine+Stress), S32006 and then subjected to stress (S32006+Stress) or Melatonin and then subjected to stress (Melatonin+Stress). Data are expressed as means ± SEM. * p < 0.01, ** p < 0.001 Newman Keuls post-hoc tests following One-way ANOVA (n = 6-7 rats/group).

After chronic drug treatments, rats were subjected to standard acute FS-stress protocol (see Methods) and, immediately after the stress session, the synaptosomes were purified from P/FC. The presynaptic release of endogenous glutamate and GABA evoked by 15 mM KCl were measured from synaptosomes in superfusion, as done previously [22,25,26]. One way ANOVA showed significant difference among groups (F_4,30 _= 6.757; p < 0.0005). Indeed, acute FS-stress increased depolarization-dependent release of endogenous glutamate from P/FC synaptosomes (45.9%) and, as previously observed with several different antidepressants [25,26], chronic agomelatine treatment completely prevented the stress-induced increase of glutamate release (Figure. 1). In the previous work [26] we also observed that agomelatine, as well as other antidepressants, did not modify glutamate release from P/FC synaptosomes in non-stressed rats (not shown here). For this reason agomelatine treatment of non-stressed rats was not replicated here; similarly melatonin or S32006 treatment of non-stressed rats was not performed here. Having agomelatine *per se *no effect on glutamate release in P/FC in the absence of stress, there was no point in testing if either of the two other compounds *per se *may modify glutamate release. As shown in Figure 1, in rats treated with melatonin and then stressed, the release of glutamate was similarly increased as in stressed rats. In rats treated with S32006 (antagonist of 5-HT_2C _receptor) a trend for reduction of stress-induced glutamate release was observed (-70%) but the compound did not significantly dampen stress-induced glutamate release. No significant changes in GABA release were induced by either FS-stress or any of the drugs. No effect of acute agomelatine treatment (same dose) was found on FS-stress increased release of endogenous glutamate.

## Discussion and Conclusion

In this study we sought to dissect the action of different receptorial components of agomelatine, with regard to the action of this drug on stress-induced glutamate release in P/FC. This was carried out by performing chronic treatments with either melatonin or a selective 5-HT_2C _receptor antagonist (S32006), both endowed with receptorial affinity comparable with the affinity of agomelatine for the respective receptors. The present results, showing that only agomelatine but not the two other compounds significantly prevented the stress-induced increase of glutamate release, suggest that the action of agomelatine on glutamate release requires both receptorial components. However, it is worth noting that while glutamate release in melatonin-treated rats was virtually indistinguishable from stressed rats, in rats treated with S32006 a trend for reduction of release was observed (-70% compared to stress group). It may be speculated that enhancement of noradrenergic and dopaminergic transmission induced by 5-HT_2C _receptor antagonism [13] in cortical areas is a necessary modification for dampening of glutamate release, similar to the outcome of treatments with monoamine reuptake inhibitors, which also increase monoamine neurotransmission with a different mechanism [for a discussion see ref. [27,28]]. However, in the case of agomelatine only the combination of melatonergic agonism with 5-HT_2C _receptor antagonism seems to be sufficient for full blockade of stress-induced glutamate release. This is in turn suggestive of a possible synergy between melatonin and 5-HT_2C _receptor-dependent pathways in the action of this drug.

These results are in line with the behavioral data published by Papp et al. [17], showing that the antidepressive effects of agomelatine in the chronic mild stress model of depression depend on a combination of its melatonin agonist and 5HT_2C _antagonist properties. Furthermore, comparable results were also obtained in the learned helplessness model of depression, where antidepressant effect was observed with agomelatine, but not after treatment with melatonin or the selective 5-HT_2C _antagonist SB242084 [14]. In this line, recently it has been demonstrated that the effects of chronic agomelatine on cell survival in hippocampus as well as on Brain Derived Neurotrophic Factor (BDNF) expression maybe due to a synergy between both properties of the drug [29,30].

Further studies, at the level of intracellular signaling and of presynaptic machinery [22,31,25,26], will investigate the suggested cross-talk between post-receptor pathways in the action of agomelatine on glutamate release.

## Methods

### Footshock stress procedure and drug treatments

Sprague-Dawley rats (170-200 gr) were purchased from Charles River (Calco, Italy). All experimental procedures were performed in accordance with the European Community Council Directive 86/609/EEC and Italian legislation on animal experimentation (Decreto Ministeriale 124/2003-A). Rats were chronically (14 days) treated with agomelatine (40 mg/kg i.p.), melatonin (40 mg/kg i.p.), S32006 (10 mg/kg i.p.), or vehicle (hydroxyethylcellulose, 1%, 1 ml/Kg, i.p.), administered at 5.00 pm (2 h before the start of the dark cycle, 7 pm). The doses of agomelatine and melatonin were selected based on their reported activities in animal models of depression [17,14], and on their effect on neurogenesis [29]. Similarly, the dose of S32006 was chosen based on previous works showing antidepressant activity on neurogenesis and BDNF [29,30], as well as on its activity on 5-HT2c receptors in vivo and on neurogenesis [32].

The FS-stress protocol was performed 16 h after the last administration, essentially as reported in ref. 33 (40-min FS-stress; 0.8 mA, 20 min total of actual shock with random intershock length between 2-8 sec). The FS-stress box was connected to a scrambler controller (LE 100-26, Panlab) that delivers intermittent shocks to the metallic floor. Sham-stressed rats (controls) were kept in the stress apparatus without delivering of shocks. Rats were killed immediately after FS-stress and P/FC were quickly dissected on ice and processed as reported below.

### Preparation of purified synaptosomes for glutamate/GABA release

Purified synaptic terminals (synaptosomes) were prepared by centrifugation on Percoll gradients [34,22] from fresh brain tissue, and resuspended in physiological medium with the following composition (mM): NaCl, 125; KCl, 3; MgSO_4_, 1.2; CaCl_2_, 1.2; NaH_2_PO_4_, 1; NaHCO_3_, 22; glucose, 10 (aeration with 95% O_2 _and 5% CO_2_); pH 7.2-7.4.

### Glutamate and GABA release experiments

Aliquots of the synaptosomal suspensions (about 100 μg protein) were layered on microporous filters at the bottom of a set of parallel superfusion chambers maintained at 37°C [35,36]. Superfusion was started at a rate of 0.5 ml/min with standard medium aerated with 95% O_2 _and 5% CO_2_. After 36 min of superfusion, to equilibrate the system, samples were collected according to the following scheme: two 3-min samples (t = 36-39 min and t = 45-48 min; basal outflow) before and after one 6-min sample (t = 39-45 min; stimulus-evoked release). A 90-sec period of stimulation was applied at t = 39 min, after the first sample has been collected. Stimulation of synaptosomes was performed with 15 mM KCl, substituting for equimolar concentration of NaCl. Fractions collected were analysed for endogenous glutamate and GABA content. Amino acid release was expressed as nmol/mg of protein. The stimulus-evoked overflow was estimated by subtracting transmitter content of the two 3-min samples (basal outflow) from release evoked in the 6-min sample collected during and after the depolarization pulse (stimulus-evoked release). Effects of drug treatments were evaluated by comparing the stimulus-evoked overflow in drug-treated animals vs. that calculated in vehicle-treated rats. Appropriate controls were always run in parallel. Endogenous glutamate and GABA were measured by high performance liquid chromatography analysis [22].

### Statistical analysis

One-way analysis of variance (ANOVA) was used for the analysis of glutamate release followed by post-hoc group comparisons with Newman Keuls test. Statistical analysis of the data was carried out using GraphPad Prism4 (GraphPad Software Inc., USA). For all analyses a value of p < 0.05 was considered statistically significant.

## Abbreviations

SSRI: Selective serotonin reuptake inhibitor; SNRI: Serotonin-norepinephrine reuptake inhibitor; FS: Footshock; P/FC: Prefrontal/frontal cortex; BDNF: Brain Derived Neurotrophic Factor.

## Authors' contributions

MP, GB and GR designed the study. MP, DT and LM wrote the ms. LM, MM, TB, MG, AM performed all the experimental work. DT performed the statistical analysis. EM, CG participated on the study design as well as on the paper discussion. All authors read and approved the final manuscript.
